# Annotations

**Published:** 1892-02-20

**Authors:** 


### Annotations.

**Universities and their Labels.**

Those who have long wished to see the capable teachers that abound in London organised into a working university, are beginning to feel some faint hope of having their wishes realised. The Bishop of London, in a letter to the Lord Mayor, puts his finger upon the weak joint in the armour of the existing London University. "The University of London," says Dr. Temple, "does excellent work in examining all comers and appraising their attainments. But the highest kind of teaching, which aims at formation of mind, cannot find free play for itself under a system which subordinates the teacher to the examiner. Such a system has a perpetual tendency to give a mechanical character both to the teaching and to its results. Originality and freshness in the teaching is killed by the perpetual necessity of paying regard, not to the subject that is to be taught, but to the examination that is to be passed." One result of the system so aptly described by the Bishop is to make "London" men value the hall-mark or label which their university puts upon them more than the culture and trained capacity of which the label is intended to be the mere indicator. It is this hall-mark which is constantly paraded by the graduates of the London University, as if it were something different from, and superior to, the labels affixed by other universities. They trust in their label to carry them successfully through all manner of difficulties and to all manner of honours and rewards. This raw state of mind operates injuriously upon the character and career of the young men who seek London degrees. The older universities, which value teaching above examination, and which, indeed, recognise that culture is as superior to the label which a university puts upon it as a prize ox is to the white rosette which proclaims his distinction to the world, avoid these foolish mistakes. The graduates of Oxford and Cambridge, if they have learnt nothing else, have learnt that the attainment of a university degree is but the very first step on the ladder of life's work and success. Whilst an Oxford or a Cambridge man is quite as proud of his distinguished university as any London man is of his, and of course with much more reason, he is well aware that he must look to himself and to his actual work and worth for the success and honour which he hopes to win in life. Teaching, and ever more teaching, is the true function of a university. When men are taught, and taught thoroughly and intelligently, the affixing of a university label upon them is a matter of quite secondary importance.

**The Lords and the London Fogs.**

Viscount Midleton rose in the House of Lords last week to ask for the appointment of a Royal Commission to deal practically with the London fogs. His lordship trotted out, not without skill, the usual facts and arguments employed by fog abolishers. The Marquis of Salisbury, in his turn, was genuinely sympathetic with the noble Viscount; but displayed in its utmost expanse the familiar banner which has "Non Possumus" for its motto. This same "Non Possumus" is a very dangerous motto indeed for an individual, for a party, for a corporation, or for a State. When men say "Non Possumus," nature is apt also to utter with all the certainty of an echo a "Non Possumus" on her own account. The simple, but absolutely inevitable truth is that we must either find healthy conditions to live in, or make them, or be destroyed in health and life for the want of them. There is no escape from this kind of solid and concrete logic. Lord Midleton reminded his fellow peers that 40,000 tons of coal are burnt in London every day, and that as a result 480 tons of sulphur, or as he might more correctly have said, of sulphurous matter, some of it much more dangerous than sulphur, are daily emitted into the atmosphere. His lordship omitted one fact, which, from a health point of view, is almost as important as our sulphurous atmosphere. This fact is that for about five months in succession every year, London is practically without sun. It may be impossible for Lord Salisbury's Government to deal effectually with the question, although we do not think it is. But it is not impossrble for scientific men to make the public understand the direful character of certain elements in the London fogs, and the deadly consequences that ensue to all sorts and conditions of men, women, and children, who dwell in London all the year round. A well-known public man, Sir Morell Mackenzie, died of a chronic chest affection in the very midst of his career the other day. Sir Morell had been partly disabled by that affection, and often made entirely miserable for years before. But it was of such a nature that if he had lived in even a moderately good climate, a score of years at least would in all probability have been added to his working life. To those who have any brains at all of a practical character, Sir Morell Mackenzie's premature death is an object lesson of a very striking sort. The plain truth is that it has come to a hand-to-hand conflict between the inhabitants of London on the one hand, and the 480 tons of sulphurous matter which they throw into the air every day on the other. If we five millions of human beings do not clear away the 480 tons of sulphur from our atmosphere, the 480 tons of sulphur will certainly clear every one of us away long before our natural time; and not only will they kill us prematurely, but they will do it in the cruellest of fashions, that of slow choking and suffocation. Besides, they make us so utterly miserable in the process. They ruin our brains: we cannot write even philosophy and history, much less novels and poetry: we cannot work and think in our scientific laboratories: our science is becoming, like ourselves, hasty, inconclusive, crude, and, to a large extent, worthless. It is an infinite pity that Lord Salisbury cannot help us. But we shall be compelled to make an effort to help ourselves. Is England, that has conquered half the world, to have all its manliness crushed out of it, to be choked and extinguished by the sulphur that rises from fires of its own making? The thing is a disgrace to us. If we submit to be smothered and killed without resistance in this way we deserve to be smothered and killed; and the sooner the better.

**The Training of Child Acrobats.**

What Mr. Benjamin Waugh has done for children generally, Miss Amye Reade seems well on the way to doing for children who are training for public performances. Whilst the soberest of men and women may find pleasure in witnessing feats of skill, activity, and strength when voluntarily performed by those who have a natural aptitude for such things, the most foolish, one would think, would be distressed and disgusted by the cruelties which are inflicted upon inapt children and young people in the course of their hard training. The *Daily Graphic* has allowed Miss Amye Reade the use of its columns to set forth certain facts on this subject which are known to herself. Some people, of course, deny that any cruelty is practised in the training of children for public performances. That is really of no moment at all. There are plenty of people who would deny that there is a sea at Dover, or a moon in the sky, if it suited their purpose to do so. Miss Amye Reade appears to have made the experiment of offering a supposititious girl for acrobatic training to a particular trainer. The supposed girl was said to be born of gentle parents, and to have been educated and refined. The trainer expressed his readiness to take the child, and to teach her her business in the approved fashion. Among other things, he said that he was prepared to administer to this little lady girl "corporal punishment, vigourously applied, and that on the right spot," if she did not get on as fast as he desired. This proposition he carefully underlined. Miss Reade says that she has herself seen at rehearsal in the ring of a circus a girl thrashed with a riding whip until it was necessary to give her brandy. One girl of 15 states that she has fainted as often as three times in one morning in the process of training, but was, to use her own words, "brought to and made to go at it again." Most of the girls suffer frightfully at this period of their training. Speaking medically, one cannot but express disgust and horror at such things. The pain that must be inflicted upon young girls when their bones, ligaments, muscles, nerves, and blood vessels are thus compelled to assume unnatural positions must be simply indescribable. Of course a civilised legislature ought not to allow anything of the kind to be done. Even parents should be forbidden to train their own children for any acrobatic performances that involve pain, injury, and distortion of limbs. It seems almost useless to appeal to a large section of the British public on questions of this kind. There are a certain number of grinning and senseless men and women who will pay for the pleasure of looking at unnatural performances; and there are a certain number of other men, who, to make money without working, would torture every child in England to death if the law would allow them. But is it for those who know better to permit such things to continue to be done? People of money and leisure must help Miss Reade in her womanly crusade against cruelty.

